# Case Report: dose adjustment of orelabrutinib for managing central nervous system post-transplant lymphoproliferative disorder following acute lymphoblastic leukemia transplantation

**DOI:** 10.3389/fimmu.2025.1597119

**Published:** 2025-06-26

**Authors:** Wenwen Wang, Jianlin Chen, Lingsu Guan

**Affiliations:** ^1^ Department of Hematology, Taizhou Central Hospital, Taizhou, China; ^2^ Department of Gastrointestinal Surgery, Taizhou Central Hospital, Taizhou, China

**Keywords:** orelabrutinib, central nervous system, post-transplant lymphoproliferative disorder, ebv, Btk inhibitor

## Abstract

We report a case of acute B-cell leukemia complicated by central nervous system (CNS) post-transplant lymphoproliferative disorder (PTLD) following allogeneic hematopoietic stem cell transplantation (allo-HSCT). The patient achieved sustained disease stabilization following therapeutic dose modification of orelabrutinib, representing the first evidence-based documentation that Bruton’s tyrosine kinase (BTK) inhibitor down-titration confers clinical efficacy in PTLD. Epstein-Barr virus (EBV)-associated PTLD is a serious complication following allogeneic HSCT, with frequent CNS involvement. Effective treatment for CNS involvement is often hampered by the challenge of drug penetration across the blood-brain barrier. This case highlights the potential benefit and safety of dose-adjusted orelabrutinib in controlling CNS PTLD, suggesting a promising therapeutic approach.

## Introduction

Post-transplant lymphoproliferative disorder (PTLD) arises from the abnormal proliferation of lymphocytes or plasma cells and exhibits marked clinical heterogeneity (1). Key post-transplant risk factors include severe graft-versus-host disease requiring strong immunosuppression, high or rising Epstein-Barr virus (EBV) levels, and mesenchymal stem cell therapy, with EBV infection being the most crucial factor (1). Management of EBV-associated central nervous system (CNS) PTLD typically involves the rituximab in combination with high-dose methotrexate (HD-MTX) and/or cytarabine-based chemotherapy, contingent upon the patient’s ability to tolerate these regimens. Additional interventions may include donor lymphocyte infusions and radiotherapy (2). Despite these treatments, 33.3% of patients died from PTLD, and no standard guidelines currently exist (1). Orelabrutinib, a selective BTK inhibitor, has shown promise for B-cell malignancies, particularly CNS lymphomas, but its efficacy and safety in PTLD, especially at lower doses, have not been established (3).

## Case report

**Figure 1:**
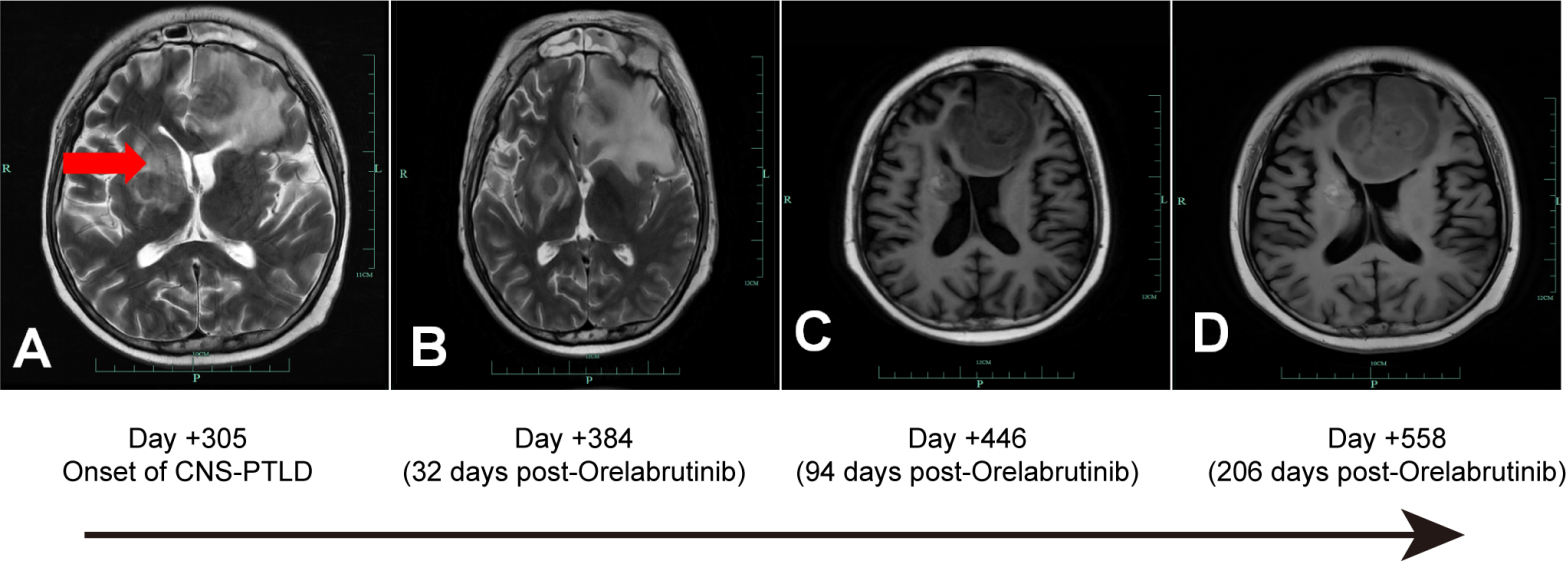
CNS-PTLD Brain MRI: **(A, B)** Axial T1-weighted post-gadolinium contrast-enhanced images; **(C, D)** Axial T2-weighted post-gadolinium contrast-enhanced images. A: Day +305 Onset of CNS-PTLD, showing a hypointense lesion in the right corpus callosum (red arrow). B: Day +384 (32 days post-Orelabrutinib): The lesion remains stable, measuring 3.14 cm at its widest point. C: Day +446 (94 days post-Orelabrutinib): The lesion and edema have decreased, with a maximum diameter of 2.30 cm. D: Day +558 (206 days post-Orelabrutinib): Further reduction in lesion size, with the maximum diameter measuring 2.0 cm.

In July 2022, a 39-year-old man from China was diagnosed with BCR-ABL-negative B-Acute Lymphoblastic Leukemia. Bone marrow analysis revealed 16.5% blasts, 74.0% immature lymphocytes, and 93.22% abnormal precursor B-cells. Karyotyping showed 46 XY, and genetic testing identified mutations in *RUNX1, SPEN, ZEB1, ATM, IDH2, RBBP6, ROBO1, NSD3*, and *CDKN2A.* On July 12, 2022, the patient began chemotherapy with the vincristine, dexamethasone, cyclophosphamide, and idarubicin (VDCP) regimen. At 0.5 months prior to transplant (M-0.5), minimal residual disease (MRD) analysis detected 1.29% abnormal precursor B-cells among nucleated cells. The patient underwent a second cycle of hyper cyclophosphamide, vincristine, liposomal doxorubicin, and dexamethasone chemotherapy at M-1. MRD analysis at M-2 was negative. Lumbar punctures with intrathecal chemotherapy on M-1 and M-2revealed no cerebrospinal fluid (CSF) abnormalities. Brain Magnetic Resonance Imaging (MRI) on M-2showed no significant anomalies. Lumbar punctures with intrathecal chemotherapy (cytarabine, methotrexate, and dexamethasone) performed at M-1 and M-2 revealed no abnormalities in CSF. Brain MRI at M-2 also showed no structural anomalies. The patient, who was seropositive for EBV IgG with undetectable serum EBV DNA, subsequently underwent haploidentical-SCT) from his father, who had the same EBV serological profile. Conditioning was administered from dayn-8 to day -1 and consisted of busulfan (0.8 mg/kg every 6 hours on days-6 to-4) and cyclophosphamide (1.8 g/m^2/day on days -3 and -2), forming the Bu/Cy-based backbone. Additional agents included cytarabine (2.0 g/m²/day on days-8 and -7), methyl-chloroethyl-cyclohexyl-nitrosourea (ACNU, 250 mg/m^2 on day –1), and anti-thymocyte globulin (ATG, total dose 6 mg/kg, administered from day -4 to -1).GVHD(Graft-versus-Host Disease) prophylaxis CSA (consisted of cyclosporine A, 2.5 mg/kg), MTX (10 mg on days +1, +3, +6, and +11), and mycophenolate mofetil (250 mg twice daily). On day +10 post-transplantation, the patient developed grade II acute cutaneous GVHD, for which ruxolitinib was initiated. Despite treatment, the rash progressed to grade III. On day +20, CSA was discontinued and replaced with recombinant human TNF receptor fusion protein plus tacrolimus, resulting in marked improvement by day +23.On day +22, the patient experienced transient neurological symptoms, including memory loss, disorientation, brief unconsciousness, jaw clenching, and limb convulsions, which resolved spontaneously within minutes. Brain MRI on day +25 revealed abnormal signals in the bilateral uncus, amygdala, and hippocampus. CSF culture identified *Micrococcus luteus* and *Moraxella osloensis*, while NGS detected *Aspergillus fumigatus* and human herpesvirus 6B (HHV-6B).Although Aspergillus fumigatus was detected by NGS in the CSF, we considered it a false positive due to the absence of pulmonary lesions on chest CT, consistently negative conventional fungal tests (G test, GM, and culture), and a single sequence read insufficient for definitive diagnosis; thus, antifungal therapy was not initiated. Antiviral therapy with foscarnet and ganciclovir, combined with valproic acid and levetiracetam for seizure control, resulted in clinical improvement. By day +48, bone marrow analysis confirmed over 99% donor-derived T, NK, and B cell chimerism. Skin GVHD improved to grade I by day +30 but relapsed on day +35 with fluctuating severity between grade II and III. Mesenchymal stem cells (5.9 × 10^7/100 mL) were administered on days +54 and +61, achieving temporary disease control. On day +63, the rash again progressed to grade III, prompting intensified immunosuppressive therapy with MTX (10 mg) and increased-dose methylprednisolone, which led to clinical improvement. Meanwhile, EBV DNA increased to 1.0 × 10^4 copies/mL and declined following basiliximab administration on days +66 and +70. A subsequent flare on day +74 was controlled with an additional MTX dose. Immunosuppressants were gradually tapered by day +263 without further recurrence. On day +305, the patient developed persistent fever. Contrast-enhanced brain MRI revealed intracranial masses with intratumoral hemorrhage and peritumoral edema in the left frontal lobe and right basal ganglia, extending into the corpus callosum (**Figure 1A**).

**Figure 2:**
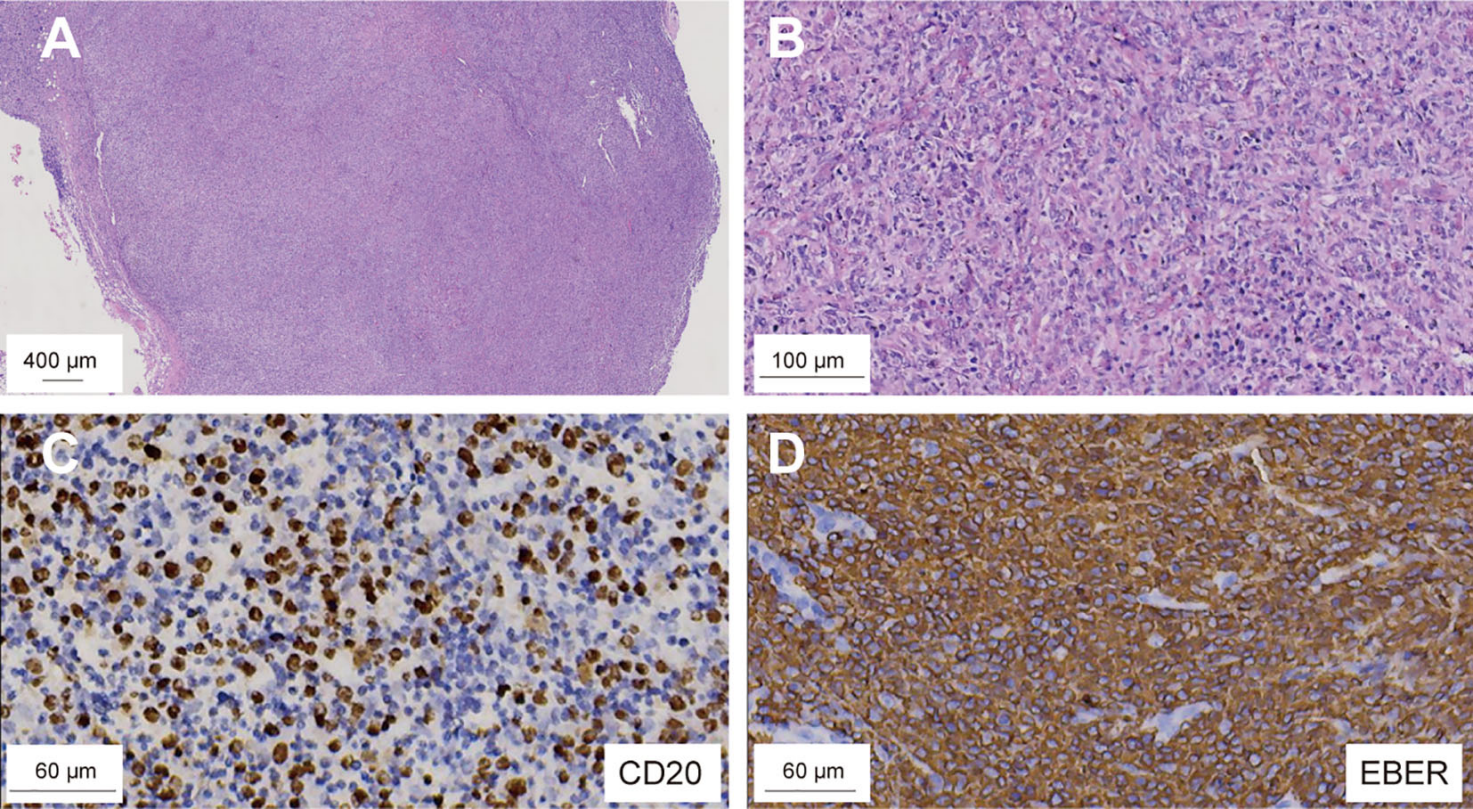
Microscopic examination of the brain biopsy reveals monomorphic DLBCL with EBV infection. H&E staining; original magnification: **(A)** ×10; **(B)** ×50. IHC shows CD20-positive infiltrating lymphocytes **(C)**, and in ISH confirms EBV-encoded RNA positivity in these cells **(D)**. DLBCL, diffuse large B-cell lymphoma; EBV, Epstein-Barr Virus; ISH, *in situ* hybridization.

Positron Emission Tomography/Magnetic Resonance Imaging (PET/MRI) revealed multiple irregular nodules and masses in the left frontal lobe and right basal ganglia, characterized by high 18F-fluorodeoxyglucose (18F-FDG) uptake, extensive edema, intratumoral hemorrhage, and compression of the anterior horns of both lateral ventricles. On day +325, a stereotactic brain biopsy showed significant lymphoid infiltration, extensive necrosis, and EBV infection, indicating immunodeficiency-associated B-cell lymphoproliferative disorder, consistent with a polymorphic stage. Immunohistochemistry was positive for CD19, CD20, CD79a, PAX5, partially positive for CD3, CD38, and Ki-67 index of 60%, but negative for CD10, CD30, CD34, CD56, TDT, MPO, Kappa, and Lambda (**Figure 2**). Epstein-Barr Virus-Encoded small RNA *in situ* hybridization (EBER-ISH) was positive, and PCR-based analysis detected immunoglobulin gene rearrangement, supporting the diagnosis of EBV-associated CNS-PTLD.

**Figure 3:**
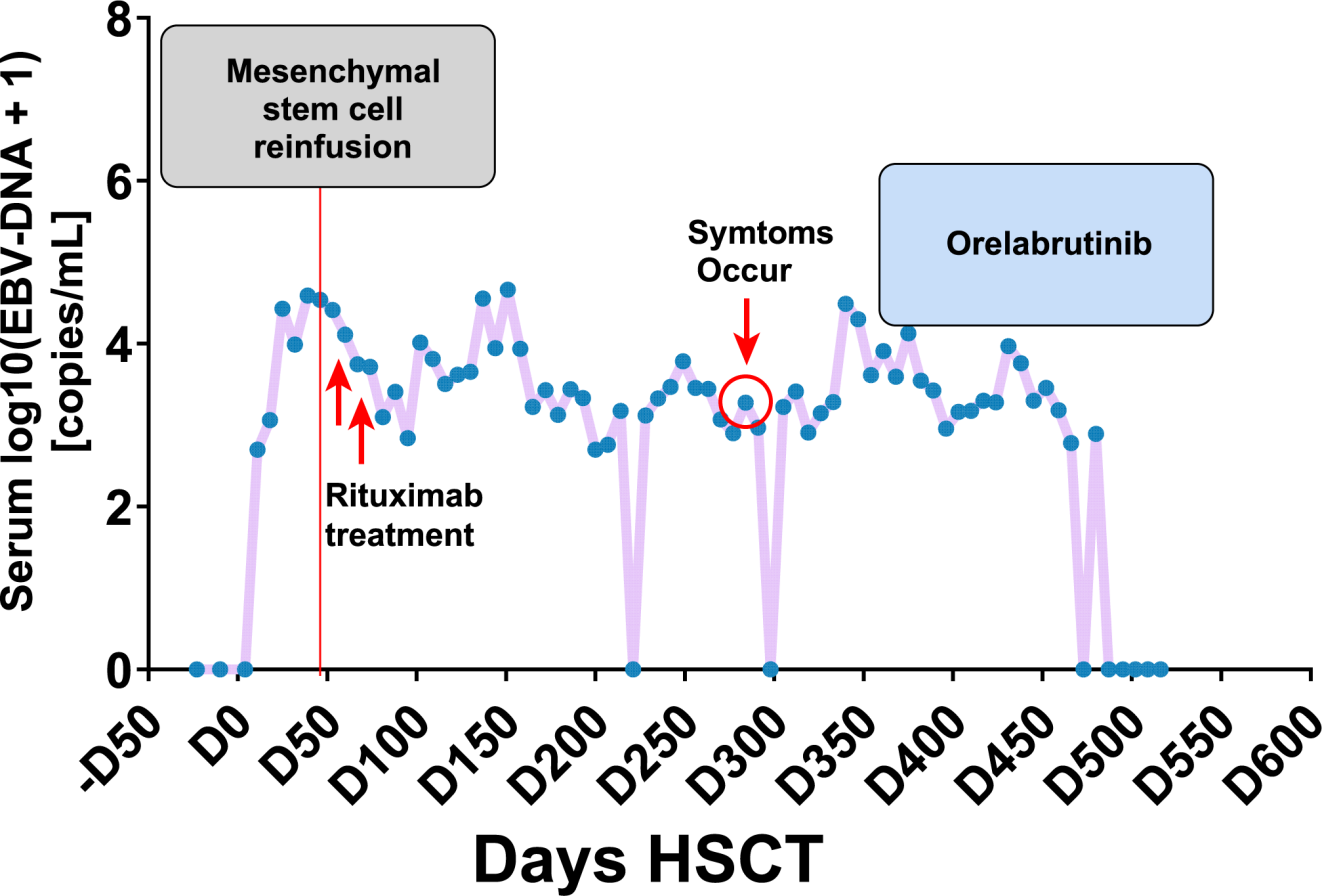
The clinical management of PTLD following transplantation, along with the corresponding fluctuations in serum EBV-DNA titers.

On day +326, the patient’s blood counts showed white blood cell (WBC) count of 1.73 ×10^9/L (normal range: 3.5-9.5 × 10^9/L), hemoglobin (Hb) of 59 g/L (normal range: 115–150 g/L), and platelets (PLTs) of 38 × 10^9/L (normal range: 125-350 × 10^9/L), with an Eastern Cooperative Oncology Group Performance Status (ECOG PS score) of 4, indicating intolerance to chemotherapy and radiotherapy. Despite supportive treatment including fluid management, hematopoietic stimulation, and anti-GVHD therapy, the patient exhibited no significant improvement in peripheral blood counts or ECOG PS. Orelabrutinib (50 mg qd) was initiated on day +352. Follow-up brain MRIs showed a sustained decrease in lesion size and surrounding edema (**Figure 1B-D**). At the last follow-up on June 19, 2024 (day +558 post-HSCT, day +206 on orelabrutinib), brain MRI showed further lesion reduction (2.0 cm), EBV DNA was undetectable in peripheral blood, and the patient was alive with an improved ECOG PS of 2(**Figure 3**). Due to thrombocytopenia and the risk of bleeding, repeat lumbar puncture was not performed.

**Figure 4:**
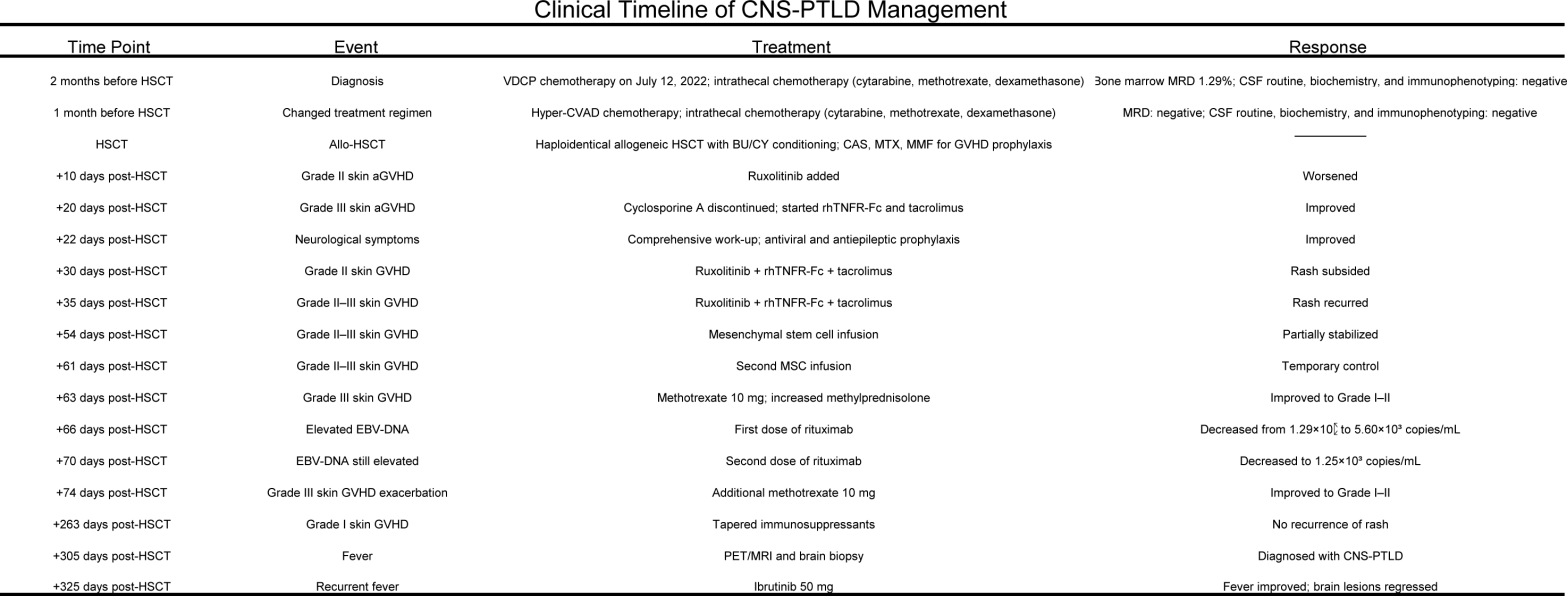
Timeline.

During therapy, WBC counts ranged from 0.88 to 8.81 × 10^9/L, PLTs from 9 to 57 × 10^9/L, and Hb from 55 to 96 g/L. Multiple enhanced cranial MRIs showed no evidence of cerebral hemorrhage. The treatment was well-tolerated, and no significant adverse events were observed. The treatment timeline and corresponding regimens are summarized in **Figure 4**.

## Discussion

CNS PTLD is a highly aggressive malignancy has a poor prognosis, with an incidence of 3.2% following allogeneic transplantation (4). A multicenter retrospective study evaluating 84 cases of primary CNS PTLD in solid organ transplant recipients reported an overall response rate of 60% and treatment-related mortality of 13% (5). At disease onset, patients exhibited marked cytopenia and an ECOG PS of 4, precluding tolerance to standard chemo-radiotherapy (5).Given prior evidence of BTK inhibitor efficacy in CNS-PTLD and the favorable CNS penetration and safety profile of low-dose orelabrutinib, reduced-dose treatment was initiated with informed consent to minimize potential toxicity. BTK inhibitors, by targeting Bruton tyrosine kinase, disrupt B-cell receptor signaling to inhibit B-cell proliferation and survival and are widely used in B-cell malignancies such as Chronic lymphocytic leukemia/small lymphocytic lymphoma and MCL (6). Orelabrutinib exhibits superior CNS bioavailability with a 10.8-fold higher median CSF concentration (21.6 ng/mL) than ibrutinib (2.0 ng/mL), supported by an enhanced CSF-to-plasma AUC 0–24 h ratio of 75-83% versus <8%, reflecting 10- to 80-fold systemic-to-CNS partitioning efficiency. CSF Cmax (7.4–10.4 nM) sustains 5-7-fold coverage above BTK IC90 potency (1.5 nM), approximately marginally meets this threshold (7, 8). In newly diagnosed Primary Central Nervous System Lymphoma, orelabrutinib combined with rituximab and HD-MTX achieved an 83.3% complete remission rate and 16.7% partial remission rate. Both case reports and trials support BTK inhibitors’ efficacy in CNS-PTLD treatment (9, 10).Reduced-dose orelabrutinib (50 mg/d) achieved rapid partial response (Lugano criteria) with undetectable dose-limiting toxicities in a thrombocytopenic patient, despite unprofiled CYP3A4 polymorphisms (AUC variability: 2.1- to 4.6-fold) (11), suggesting compensatory metabolic adaptation beyond conventional pharmacogenomic predictors. Preclinical evidence of persistent mTORC1-driven tumor viability (62% vs vehicle) (12) raises concerns that subtherapeutic BTK inhibition may paradoxically accelerate adaptive resistance via PI3K/AKT pathway hyperactivation, necessitating longitudinal clonal dynamics monitoring in dose-de-escalated regimens.

## Conclusion

Reduced-dose orelabrutinib may provide a viable treatment option for PTLD in post-ALL transplant patients. These findings warrant further investigation through mechanistically oriented preclinical models and prospectively designed clinical trials to establish translational validation while defining the optimal dosing strategy that balances sustained efficacy with hematologic safety profiles.

## Data Availability

The original contributions presented in the study are included in the article/supplementary material. Further inquiries can be directed to the corresponding author/s.
